# Nerve Injury-Induced c-Jun Activation in Schwann Cells Is JNK Independent

**DOI:** 10.1155/2014/392971

**Published:** 2014-04-28

**Authors:** Charlotta Lindwall Blom, Lisa B. Mårtensson, Lars B. Dahlin

**Affiliations:** ^1^Cellectricon AB, Flöjelbergsgatan 8C, 431 37 Mölndal, Sweden; ^2^Department of Biology/Functional Zoology, Lund University, Sölvegatan 35, 223 62 Lund, Sweden; ^3^Department of Clinical Sciences in Malmö/Hand Surgery, Lund University, Skåne University Hospital, Jan Waldenströms Gata 5, 205 02 Malmö, Sweden

## Abstract

We investigated (a) if activation of the mitogen activated protein kinase (MAPK) pathway was linked to the stress activated protein kinase (SAPK) pathway and (b) if JNK was required for activation of c-Jun in Schwann cells of rat sciatic nerve following injury. To this aim, ERK1/2 and the transcription factors c-Jun and ATF-3 were studied by immunohistochemistry in segments of transected nerves. We utilized pharmacological inhibitors of both signal transduction pathways *in vitro* to determine the effects on downstream signalling events, such as c-Jun activation, and on Schwann cell survival and proliferation. A transection induces *c-Jun* and *ATF-3* transcription in Schwann cells. These events are followed by Schwann cell activation of c-Jun in the injured nerve. The MAPK inhibitor U0126 blocked ERK1/2 activation and reduced Schwann cell proliferation as well as induction of *c-Jun* transcription. The JNK inhibitor SP600125 reduced Schwann cell proliferation, but did not affect the expression of ERK1/2 or injury-induced increases in c-Jun or ATF-3 levels. Importantly, nerve injury induces Schwann cell activation of c-Jun by phosphorylation, which, in contrast to in sensory neurons, is JNK independent. MAP kinases, other than JNK, can potentially activate c-Jun in Schwann cells following injury; information that is crucial to create new nerve reconstruction strategies.

## 1. Introduction

Nerve injuries are difficult to treat and the outcome of surgery may be frustrating both for the patient and for the surgeon. In order to develop new treatment strategies, the understanding about the delicate mechanisms that orchestrate the nerve regeneration process has to be deepened and such knowledge is crucial also for the surgeon that repairs and reconstructs nerve injuries. Different signalling pathways are activated in cells after injury with the purpose of initiating the nerve regeneration process. The mitogen activated protein kinase (MAPK) ERK1/2 (extracellular signal-related kinase) and the stress activated protein kinase (SAPK) c-Jun N-terminal kinase (JNK) are examples of pathways that are activated by nerve injury in both neurons and Schwann cells (SCs) [1–4]. JNKs are activated most potently by inflammatory cytokines and a variety of chemical and radiant stress conditions. JNK is encoded by the* JNK1*,* JNK2,* and* JNK3* genes [5–8], and ten different JNK isoforms have been identified [5–7, 9].

Myelinating SCs express the transcription factor c-Jun, a specific JNK target, following transection of a peripheral nerve [10]. JNK mediates activation of c-Jun, which is followed by the nuclear translocation of ATF-3 [11], the latter being a member of the ATF/CREB subfamily of bZip transcription factors [12–14]. ATF-3 is induced by various signals, such as cytokines, nerve growth factor depletion, and oxidative stress, and the JNK/SAPK pathway plays an important role in induction of* ATF-3* transcription [15].

Others and we have shown that the transcription factor c-Jun is activated by JNK-mediated phosphorylation and both c-Jun and ATF-3 are upregulated in neurons and SCs after nerve injury [12, 14, 16, 17]. In dorsal root ganglia (DRG) neurons, JNK inhibition blocks c-Jun activation and ATF-3 induction with concomitant inhibition of axonal outgrowth [11]. However, the impact of these transcription factors on SC proliferation and other injury-associated events, such as survival and cell death, has yet to be investigated. We have, however, previously shown that ERK1/2 is activated in SC at the site of a nerve injury. Furthermore, inhibition of the activation of ERK1/2 significantly reduces the number of proliferating SCs [18].

In this study, we raised the question of whether ERK1/2 activation could be linked to the SAPK pathway and whether JNK was required for activation of c-Jun in SCs in a manner similar to that observed in sensory neurons [11]. We also wanted to determine the roles of these pathways in SC survival and proliferation in the injured nerve. In order to answer these questions, we studied signal transduction in SCs in response to a nerve injury in the rat sciatic nerve with focus on the activation and upregulation of signalling molecules in the MAP- and SAP-kinase pathways. In this context, our results illustrate that sciatic nerve axotomy triggers a chain of events. First, c-Jun, which is present in the SC nuclei at the time of the injury, is activated. Such activation triggers transcription of the* c-Jun* and* ATF-3* genes, followed by a second wave of c-Jun activation, where newly transcribed c-Jun is phosphorylated. The MAPK inhibitor U0126 blocked ERK1/2 activation and reduced SC proliferation and also the upregulation of c-Jun. The JNK inhibitor SP600125 reduced SC proliferation but did not have any effect on ERK1/2, c-Jun, or ATF-3 induction in the SCs. Knowledge about these mechanisms is an example of steps in translational research in nerve injury and repair.

## 2. Materials and Methods

### 2.1. Animals

Adult female Sprague-Dawley rats (Mollegaard, Denmark) were used in all experiments. The ethical committee on experimental animals in Lund, Sweden, approved the experimental procedures. The animals were kept on a 12/12 h light/dark cycle with water and food* ad libitum*.

### 2.2. *In Vivo* Experiments

Rats were anesthetised with an intraperitoneal (i.p.) injection of 0.25 mL mixture of diazepam (5 mg/mL) (Alpharma, Denmark), sodium pentobarbital (60 mg/mL) (Apoteksbolaget, Sweden), and 0.9% NaCl (2 : 1 : 1 volume proportions). The right sciatic nerve was exposed at midthigh level and transected, while the contralateral sciatic nerve was exposed but was not transected. The wounds were closed with sutures and the animals were allowed to recover for specific periods of time.

All animals were sacrificed by an i.p. overdose of sodium pentobarbital (60 mg/mL) (Apoteksbolaget, Sweden) followed by heart puncture. The sciatic nerve was exposed bilaterally and segments proximal and distal to the transection site on the experimental nerve, as well as their contralateral counterparts, were dissected and fixed in Stefanini's fixative (4% paraformaldehyde, 0.03% saturated picric acid in 0.1 M phosphate buffered saline (PBS)), overnight (o.n.). They were then washed for 3 × 20 min in PBS and cryoprotected in 20% sucrose in PBS overnight. The nerve pieces were frozen in Tissue-Tek (Sakura, Japan) and sectioned longitudinally on a cryostat at a thickness of 10 *μ*m. Sections were collected on poly-L-lysine coated microscope slides (Menzel, Germany) and stored at −20°C before further processing (see Immunohistochemistry).

### 2.3. *In Vitro* Experiments: Organotypic Culture of Sciatic Nerve Segments

The sciatic nerves on both sides were dissected and cut into 4 mm long pieces. The pieces were placed in culture dishes containing serum-free RPMI-1640 medium supplemented with penicillin/streptomycin (Invitrogen, UK). The nerve segments were incubated at 37°C for specific time points from 10 min to 48 h.

Separate nerve segments were cultured for either 2 h or 48 h in the presence of the MAPK inhibitor U0126 (Alexis Corporation, USA) or the SAPK inhibitor SP600125 (Calbiochem, Germany) at final concentrations of 5 *μ*M and 10 *μ*M, respectively. To verify the specificity of the SAPK inhibitor, explanted L4 and L5 DRG were cocultured with the nerve segments for 48 h. To examine SC proliferation, the thymidine analogue and cell cycle marker bromodeoxyuridine (BrdU) (Sigma, Sweden) was added to cultures at a final concentration of 55 *μ*M after 24 h of culturing. In these experiments, the preparations were incubated for an additional 24 h before the nerve pieces were fixed and sectioned as described above.

### 2.4. Immunohistochemistry

For immunohistochemical detection, sections were washed for 3 × 5 min with PBS (used for all washing steps) and incubated overnight at 4°C with specific antibodies (Table 1). All antibodies were diluted in PBS containing 0.25% BSA and 0.25% Triton X-100. The sections were then washed, followed by incubation for 1 h in room temperature (RT) with secondary antibodies (Table 1). The sections were then washed again and incubated for 3 min with the nuclear counterstain bisbenzimide (1 : 10000 in PBS). Finally, the sections were washed and coverslipped with PBS/glycerol (1 : 1).

For detection of BrdU, the sections were washed for 3 × 5 min with PBS, incubated for 10 min with 0.5% Triton X-100 in PBS, and washed again and incubated with 2 M HCl for 1 h at 37°C. After washing and blocking with 0.5% dry-milk in PBS for 30 min, the sections were incubated overnight at 4°C with a primary mouse-anti-BrdU antibody (DAKO, Denmark) diluted 1 : 50 in PBS with Triton X-100. After washing, the sections were incubated for 1 h with a goat-anti-mouse AlexaFluor 594 antibody (Molecular Probes, USA) diluted 1 : 320 in PBS with Triton X-100. Nuclei were counterstained as described above and the sections were coverslipped in PBS/glycerol (1 : 1). As an example of co-localization of the different markers, a double staining with ATF-3 and S-100 was performed as previously described [19].

### 2.5. Photography and Image Analysis

Images were captured using an Olympus AX70 fluorescence microscope (Olympus, Japan) equipped with a Nikon DS-Ri1 camera and the NIS-Elements BR3.0 image acquisition programme (Nikon, Japan). The images were converted to 8-bit greyscale TIFF using Adobe Photoshop 9.0.2 (Adobe, USA) and imported into ImageJ 1.40 g (a public domain image analysis program developed at the US National Institutes of Health and available on the Internet at http://rsb.info.nih.gov/nih-image/). The ImageJ tool* Threshold* was used to determine the immunopositive area of the nerve sections. The image analysis was performed on images captured using the 10x objective.

A region of interest (ROI; 100 × 100 pixels) was selected in the endoneurial area furthest away from the transection site in order to estimate the background labelling intensity. To determine immunolabeling, the threshold was then set to ±3 standard deviations of the background level. The immunopositive area was then measured on the entire section and expressed in percent of the total area of the nerve section. The number of immunopositive and bisbenzimide-positive nuclei was quantified using the tool ImageJ tool* Particle Analysis*, where the minimum and maximum particle sizes were set to 20 and 200 pixels, respectively, using the 10x objective. In all cases, the number of immunopositive nuclei is expressed as a percentage of the total number of nuclei in the sciatic nerve sections.

If not otherwise indicated, a total of five sections per time point and section site (distal, proximal, and contralateral control; number of nerves = 5) were analysed for immunoreactivity* in vivo* and a total of six sections per time point (number of nerves = 6) were analysed in the* in vitro* experiments. In the DRG and control experiments, the number of specimens analysed was *n* = 3.

### 2.6. Statistics

Analysis of variance (ANOVA) was used to determine the significance of the injury-induced immunoreactivity. The Mann-Whitney *U* test (MW) was used to evaluate if there were any significant changes in immunoreactivity after nerve segment treatment with inhibitors. The software used for the statistical analyses was StatView 5.0.1 (SAS Institute Inc., USA).

## 3. Results

### 3.1. *In Vivo* Sciatic Nerve Axotomy

#### 3.1.1. c-Jun

**Figure 1:**
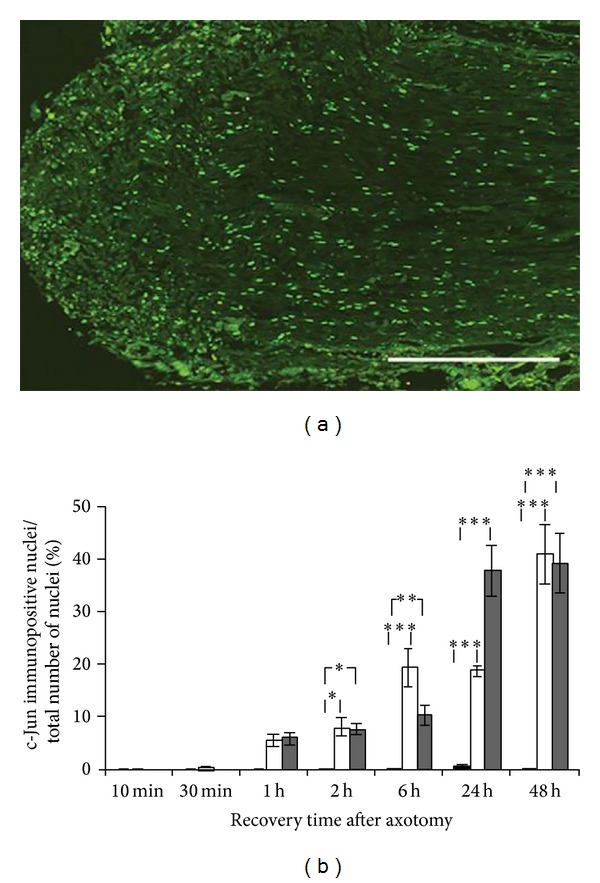
Induction of c-Jun in the axotomized sciatic nerve. (a) c-Jun immunopositive nuclei in the distal rat sciatic nerve segment* in vivo* 48 h after axotomy (to the left on the section). Scale bar = 500 *μ*m. (b) c-Jun immunoreactivity 10 min to 48 h after axotomy* in vivo*. The immunopositive nuclei are expressed as a percentage of the total number of nuclei in the nerve sections. Black bars show contralateral control nerve segments, white bars show proximal nerve segments, and grey bars show distal nerve segments. Mean values ± SEM, *n* = 5. *P < 0.05, **P < 0.01, and ***P < 0.001.

After sciatic nerve axotomy, c-Jun was gradually upregulated both distal and proximal to the site of transection (Figures 1(a) and 1(b)). The c-Jun immunoreactivity was found in SC nuclei throughout the whole nerve segment section (Figure 1(a)). The increase in c-Jun immunoreactivity was first visible at 1 h and continued to increase for up to 48 h both distal and proximal to the lesion. At 2 h, the c-Jun immunoreactivity in the injured nerve was significantly higher in both the proximal (*P* = 0.016) and distal (*P* = 0.022) segments compared to the control nerve. At 48 h, the number of c-Jun positive SC nuclei had increased significantly (*P* < 0.0001) for both the proximal and distal sciatic nerve segments as compared to the control.

#### 3.1.2. pc-Jun

**Figure 2:**
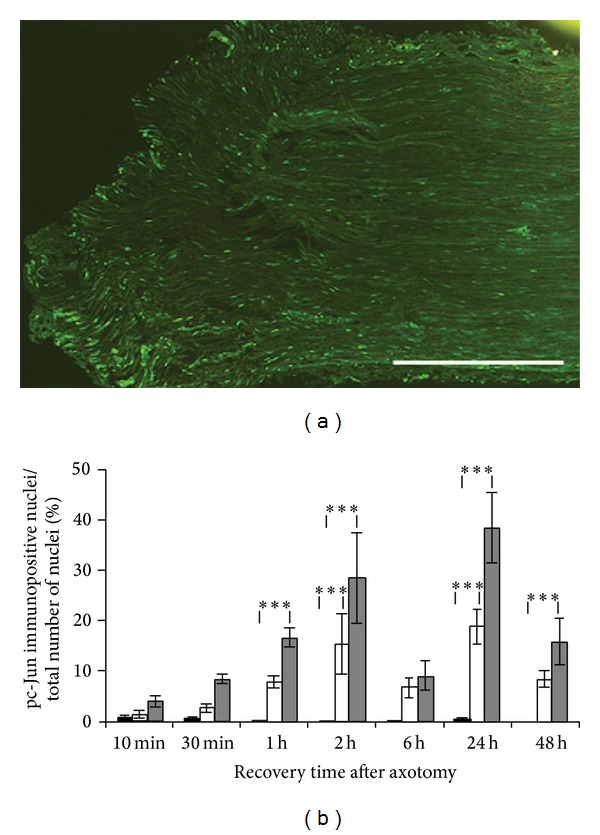
Activation of c-Jun following sciatic nerve axotomy. (a) pc-Jun immunopositive nuclei in the proximal rat sciatic nerve segment* in vivo* 2 h after axotomy (to the left on the specimen). Scale bar = 500 *μ*m. (b) pc-Jun immunoreactivity 10 min to 48 h after axotomy* in vivo*. The immunopositive nuclei are expressed as a percentage of the total number of nuclei in the nerve sections. Black bars show contralateral control nerve sections, white bars show proximal nerve segments, and grey bars show distal nerve segments. Mean values ± SEM, *n* = 5. ***P < 0.001.

The number of pc-Jun immunopositive nuclei increased as early as 10 minutes after axotomy and continued to increase for up to 2 h but then decreased again at 6 h (Figures 2(a) and 2(b)). At 1 h, the increase in immunoreactivity was significant in the distal segment compared to the control (*P* = 0.0004), and at 2 h, both the proximal and distal segments displayed a significant increase in number of pc-Jun positive nuclei (*P* = 0.0009 and *P* < 0.0001, resp.). At 6 h, there was no significant difference in the number of pc-Jun immunopositive SC nuclei as compared to in the control sections, but this was followed by a new increase at 24 h (*P* < 0.0001 for both proximal and distal segments) followed by another decline at 48 h. At 48 h, however, the number of immunopositive nuclei in the distal nerve segment was still significantly higher than in the control nerve (*P* = 0.0007). The pc-Jun immunoreactivity was mainly observed in SC nuclei in proximity to the transection site, but some positive nuclei were dispersed along the entire section (Figure 2(a)).

#### 3.1.3. ATF-3

**Figure 3:**
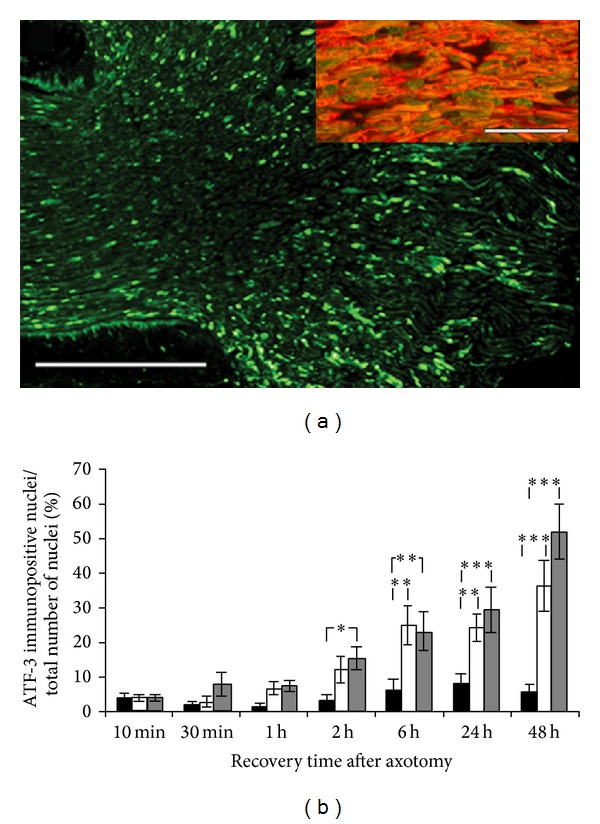
ATF-3 is upregulated in sciatic nerve Schwann cells following axotomy. (a) ATF-3 immunopositive nuclei in the distal rat sciatic nerve segment* in vivo* 48 h after axotomy (to the left on the specimen). Scale bar = 500 *μ*m. The insert shows double staining of ATF-3 (green) and S-100 (red); scale bar = 100 *μ*m. (b) ATF-3 immunoreactivity 10 min to 48 h after axotomy* in vivo*. The immunopositive nuclei are expressed as a percentage of the total number of nuclei in the nerve sections. Black bars show contralateral control nerve segments, white bars show proximal nerve segments, and grey bars show distal nerve segments. Mean values ± SEM, *n* = 5. *P < 0.05, **P < 0.01, and ***P < 0.001.

ATF-3 was upregulated in increasing numbers of SC nuclei following sciatic nerve transection* in vivo* both distal and proximal to the transection site (Figures 3(a) and 3(b)). The ATF-3 immunoreactivity was found in SC nuclei, as indicated by double staining with ATF-3 and S-100 (insert in Figure 3(a)), throughout the whole nerve section, although the numbers were at their highest close to the transection site (Figure 3(a)). The numbers of ATF-3 immunopositive nuclei had increased significantly in the distal nerve segment at 2 h, compared to in the control (*P* = 0.04), and this increase was continuous from 6 h for up to 48 h both proximal and distal to the lesion (*P* < 0.0001).

### 3.2. Organotypic Sciatic Nerve Cultures

In order to manipulate and control the environment of the injured nerve and to perform experiments with inhibitors, transected sciatic nerve pieces were cultured* in vitro*. We have previously shown that, with respect to proliferation, SCs respond to these conditions in the same way as they do following* in vivo* axotomy [18]. The present experiments confirm and extend those previous findings.

#### 3.2.1. c-Jun

**Figure 4:**
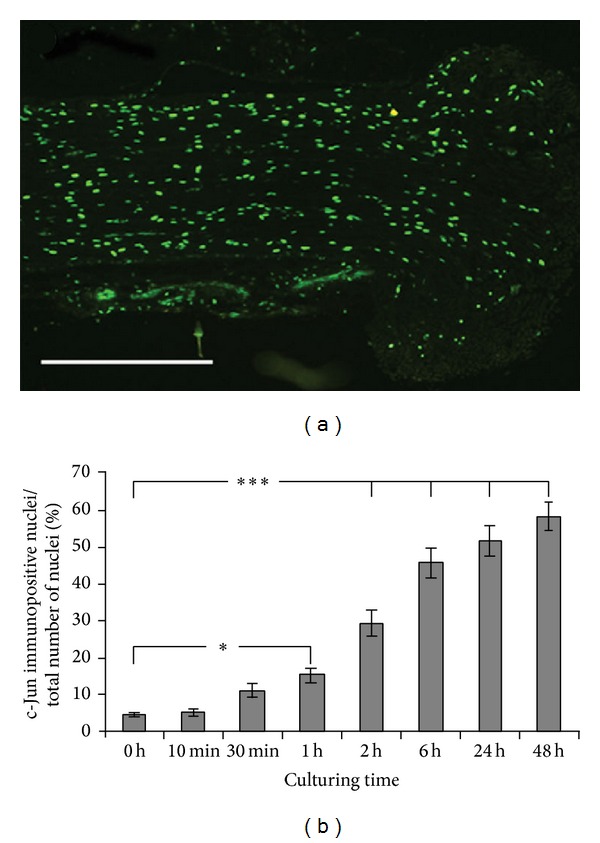
Induction of c-Jun in Schwann cells in the* in vitro* cultured sciatic nerve. (a) c-Jun immunopositive areas in the rat sciatic nerve after 48 h of culturing (edge of nerve to the right). Scale bar = 500 *μ*m. (b) c-Jun immunoreactivity in rat sciatic nerve sections after culturing for 10 min to 48 h* in vitro*. The immunopositive nuclei are expressed as a percentage of the total number of nuclei in the nerve sections. Mean values ± SEM, *n* = 6. *P < 0.05, and ***P < 0.001.

In cultured pieces of the sciatic nerve, c-Jun was upregulated in SC nuclei throughout the length of the nerve section (Figure 4(a)). The c-Jun upregulation became visible at 30 min after dissection and the upregulation increased continuously for up to 48 h (Figure 4(b)). After 1 h of culturing, the increase in numbers of c-Jun immunopositive SCs was significantly higher than in the control nerve section (*P* = 0.011), and after 2 h, the relative number of c-Jun immunopositive cells had increased 6-fold (*P* < 0.0001).

#### 3.2.2. pc-Jun

**Figure 5:**
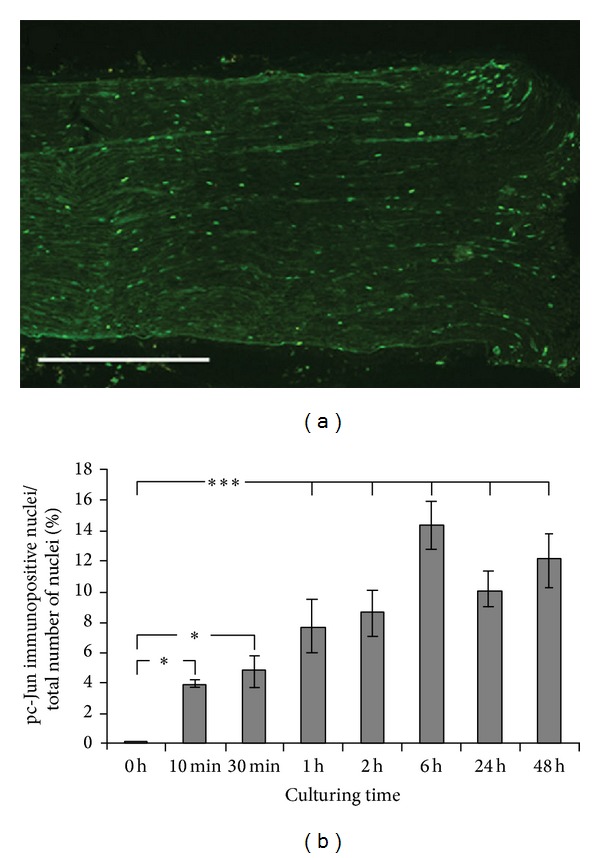
c-Jun in Schwann cells is activated by phosphorylation following* in vitro* axotomy. (a) pc-Jun immunopositive areas in the rat sciatic nerve after 6 h of culturing (edge of nerve to the right). Scale bar = 500 *μ*m. (b) pc-Jun immunoreactivity in rat sciatic nerve sections after culturing for 10 min to 48 h* in vitro*. The immunopositive nuclei are expressed as a percentage of the total number of nuclei in the nerve sections. Mean values ± SEM, *n* = 6. *P < 0.05, and ***P < 0.001.

In cultured sciatic nerve segments, increasing numbers of SCs displayed pc-Jun immunoreactivity from as early as 10 min after dissection to reach its peak at 6 h (*P* = 0.04 for 10 min and *P* < 0.0001 for 6 h compared to the control). This early increase was then followed by a decrease over the next few hours (*P* = 0.024 for the difference in pc-Jun immunopositive SC nuclei between 6 h and 24 h; Figures 5(a) and 5(b)). However, the number of immunopositive SC nuclei in the cultured nerve segments, compared to control sections, was still significant at 24 and 48 h (*P* < 0.0001). No significant difference in the numbers of pc-Jun positive SC nuclei between 24 and 48 h was however observed (*P* = 0.301). The pc-Jun immunoreactivity was found in the SC nuclei in high numbers close to the transection site and in fewer numbers dispersed along the length of the nerve segment (Figure 5(a)).

#### 3.2.3. ATF-3

**Figure 6:**
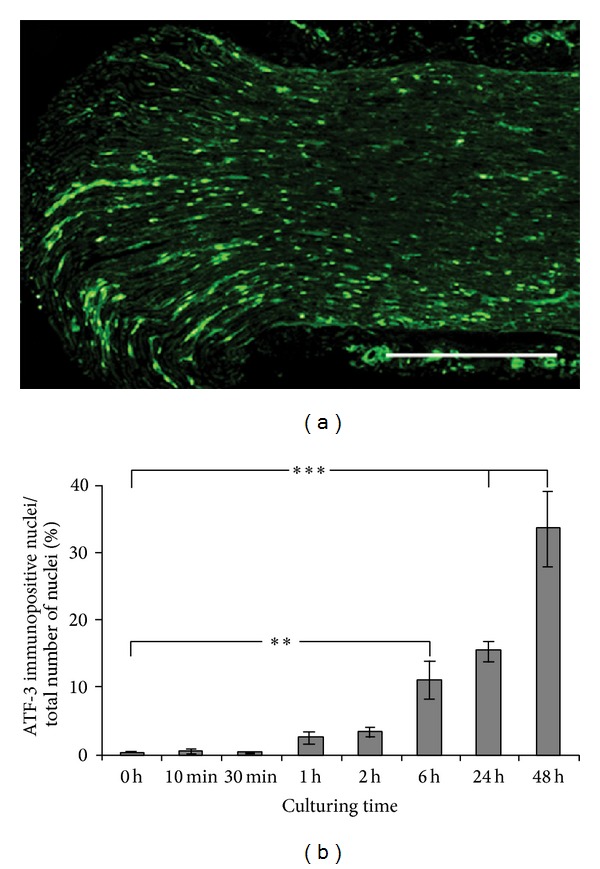
ATF-3 is induced in SCs by sciatic nerve axotomy* in vitro.* (a) ATF-3 immunopositive areas in the rat sciatic nerve after 48 hours of culturing (edge of nerve to the left). Scale bar = 500 *μ*m. (b) ATF-3 immunoreactivity in rat sciatic nerve sections after culturing for 10 min to 48 h* in vitro*. The immunopositive nuclei are expressed as a percentage of the total number of nuclei in the nerve sections. Mean values ± SEM, *n* = 6. **P < 0.01, and ***P < 0.001.

ATF-3 was upregulated over time in SC nuclei throughout the length of the cultured sciatic nerve segments, although the highest numbers were observed close to the transection site (Figure 6(a)). The increase in ATF-3 immunoreactivity was first visible at 1 h after dissection and then continued for up to 48 h (Figure 6(b)). At 6 h, the number of ATF-3 immunopositive SCs had increased significantly as compared to the control (*P* < 0.0018) and the increase was still significant at 24 and 48 h (*P* < 0.0001).

#### 3.2.4. Effects of the MAPK Inhibitor

**Figure 7:**
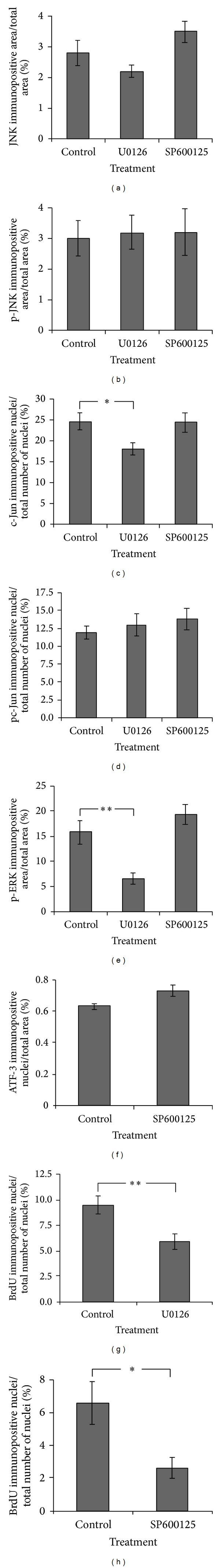
Inhibition of MAPK and SAPK in the organotypic sciatic nerve cultures. The effect of the MAPK inhibitor U0126 and the SAPK inhibitor SP600125 on nerve pieces cultured for either 2 h ((a), (b), (c), (d), and (e)) or 48 h ((f), (g), and (h)). The graphs show JNK (a), p-JNK (b), and p-ERK1/2 (e) immunopositive area and c-Jun (c), pc-Jun (d), and ATF-3 (f) immunopositive nuclei. Error bars = mean values ± SEM, *n* = 6 ((a)–(f)), *n* = 5 ((g) and (h)). *P < 0.05, and **P < 0.01.

In sciatic nerve segments that had been treated with the MAPK inhibitor U0126, the upregulation of c-Jun was significantly reduced (*P* = 0.0158), but this treatment had no inhibitory effect on activation of c-Jun (i.e., the number of pc-Jun immunopositive SCs) (Figures 7(a)–7(d)). The phosphorylation of ERK1/2 in the SC cytoplasm and also BrdU incorporation were blocked by U0126 (*P* = 0.0018 for p-ERK1/2 and *P* = 0.0077 for BrdU) in accordance with our previous results [18] (Figures 7(e) and 7(g)).

#### 3.2.5. Effects of the SAPK Inhibitor

In the present experiments, SP600125 inhibited SC BrdU incorporation in the organotypic sciatic nerve cultures (*P* = 0.022) (Figure 7(h)). However, no effect on the immunoreactivity of c-Jun, pc-Jun, ATF-3, or ERK1/2 in the SCs was observed (Figures 7(a)–7(f)).

**Figure 8:**
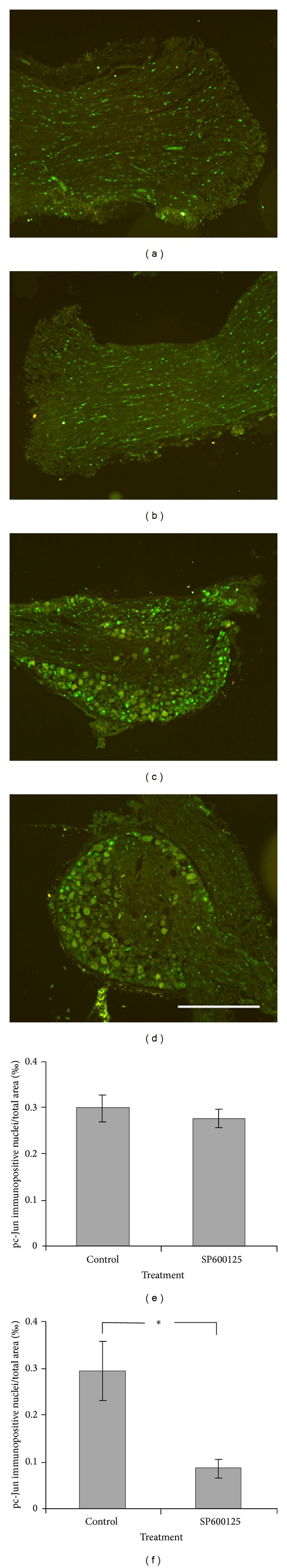
The effect of the SAPK inhibitor SP600125 on c-Jun activation in sciatic nerve segments and in DRGs cultured for 48 h. pc-Jun immunoreactivity in a nerve piece (edge of nerve segments to the right (a) and left (b)) cultured without the inhibitor (a) and with the inhibitor (b). pc-Jun immunoreactivity in a DRG cultured without the inhibitor (c) and with the inhibitor (d). The graphs show pc-Jun immunopositive SC nuclei as ‰ of the total area of the nerve section (e) and pc-Jun immunopositive nerve cell nuclei as ‰ of the total area of the section of the DRG (f). Error bars = mean values ± SEM, *n* = 3. *P < 0.05.

**Figure 9:**
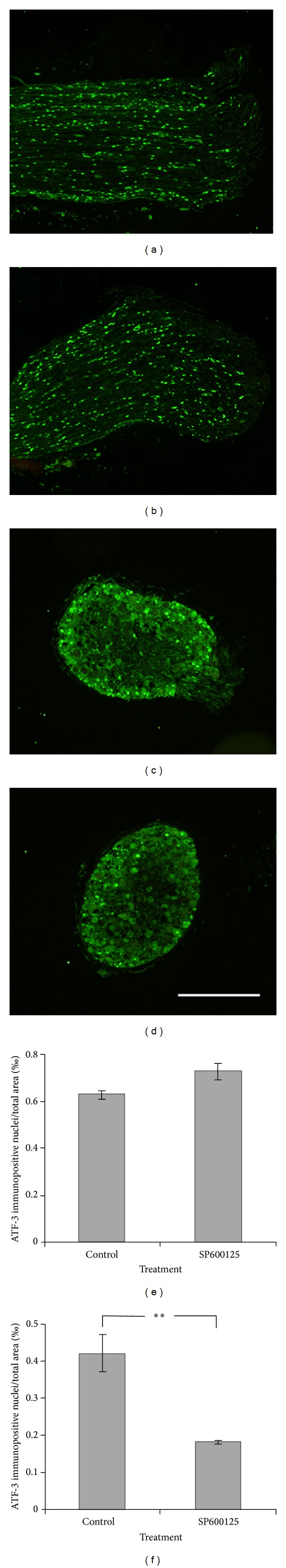
The effect of the SAPK inhibitor SP600125 on ATF-3 upregulation in sciatic nerve segments and DRGs cultured for 48 h. ATF-3 immunoreactivity in a nerve piece (edge of nerve segments to the right) cultured without the inhibitor (a) and with the inhibitor (b). ATF-3 immunoreactivity in a DRG cultured without the inhibitor (c) and with the inhibitor (d). The graphs show ATF-3 immunopositive SC nuclei as ‰ of the total area of the nerve section (e) and pc-Jun immunopositive nerve cell nuclei as ‰ of the total area of the section of the DRG (f). Error bars = mean values ± SEM, *n* = 3. **P < 0.01.

Since these results were very different from previous experiments on DRG sensory neurons [17], control experiments were made to ensure that the inhibitor was not defective or improperly handled. Therefore, we co-cultured nerve segments and DRG and treated them with the SP600125 inhibitor. In accordance with our previous results, we found that the SAPK inhibitor had no effect on the activation of c-Jun (Figures 8(a), 8(b), and 8(e)), or on the upregulation of ATF-3 (Figures 9(a), 9(b), and 9(e)) in the sciatic nerve segments. However, in the DRG, both c-Jun activation (Figures 8(c), 8(d), and 8(f)) and ATF-3 (Figures 9(c), 9(d), and 9(f)) upregulation were reduced (*P* = 0.0304 for pc-Jun and *P* = 0.0093 for ATF-3) in neuronal nuclei, confirming that the SP600125 inhibitor was working. However, this result indicated that separate signalling mechanisms are operative in neurons and Schwann cells following transection of the sciatic nerve.

## 4. Discussion

Here we report that a sciatic nerve injury gives rise to c-Jun activation followed by an upregulation of both c-Jun and ATF-3 in SCs within the nerve. Interestingly, using MAPK and SAPK inhibitors, we also demonstrate that c-Jun activation is JNK independent in the SCs of the rat sciatic nerve. This novel discovery represents the principal finding of the present study, which, for the first time, illustrates that activation of c-Jun is differently regulated in SCs compared to in DRG sensory neurons.

In DRG neurons, activation of c-Jun and induction of ATF-3 are events, which are associated with survival and axonal outgrowth following a peripheral nerve injury [11]. The present study shows that c-Jun activation also occurs in SCs within the nerve following a sciatic nerve injury and that this activation is associated with Schwann cell survival and proliferation. In SCs, we found that c-Jun was upregulated as a response to nerve injury following sciatic nerve transection in both* in vivo* and* in vitro* cultured nerve segments. Induction of* c-Jun* transcription in SCs was, however, preceded by activation of already existing c-Jun through phosphorylation, which could be observed in the SC nuclei as early as 10 minutes after the nerve injury. The time course of c-Jun activation and induction is consistent with the suggestion that c-Jun regulates its own transcription [20]. The numbers of c-Jun- and pc-Jun-stained SCs were initially, that is, within 2 h, somewhat different. We have no clear explanation, but the differences were rather small and the extent of SD (i.e., SEM in figures) may contribute to the discrepancy and the explanation. ATF-3 was also rapidly upregulated in the transected nerve as a response to the injury and was observed in SC nuclei close to the transection site, but also along the length of the nerve. We have previously established a connection between activation of c-Jun and the induction of ATF-3 in sensory neurons, where ATF-3 is associated with cell survival and regeneration [17]. In addition, it has been shown that ATF-3 expression in both SCs and neurons is important for axonal outgrowth after a peripheral nerve repair with respect to the timing of such a procedure [21, 22]. Our results therefore further indicate that Schwann cells in the injured peripheral nerve are involved in the early repair process, a clinically important aspect.

The possible relationship between the MAPK and SAPK pathways was investigated by the use of inhibitors of these signalling pathways* in vitro*. As expected, the ERK1/2 inhibitor U0126 effectively suppressed ERK1/2 activation and reduced SC proliferation [18]. Although U0126 had no inhibitory effect on c-Jun activation, it reduced c-Jun upregulation in the sciatic nerve, indicating a link between the ERK1/2 and JNK pathways at the level of SC transcription. The effect on c-Jun induction is in accordance with the findings of Clerk et al. [23, 24], who demonstrated that ERK1/2 is involved in the upregulation of c-Jun in cardiac myocytes. A coupling between ERK1/2 and c-Jun is also indicated by the findings that c-Jun activation in the transected sciatic nerve exhibited a biphasic response, that is, the same pattern as that of ERK1/2 activation in our previous study [18].

The JNK inhibitor SP600125 inhibited SC proliferation in the transected sciatic nerve, lending additional evidence to the suggestion that the JNK pathway is involved in the regulation of SC proliferation. We believe this to be a very interesting result, which, however, requires further investigation. Although the SP600125 inhibitor efficiently suppresses activation of c-Jun and the upregulation of ATF-3 in sensory neurons [11], it did not inhibit neither c-Jun activation nor ATF-3 upregulation in SCs. Thus, there is a major difference between neurons and SCs following nerve injury with respect to regulation of c-Jun activation and the upregulation of ATF-3. These results further corroborate the suggestion that MAP kinases other than JNK have the potential to activate c-Jun in glial cells. Therefore, we suggest that a JNK-independent activation of c-Jun occurs in Schwann cells following a peripheral nerve injury. Such JNK-independent activation of c-Jun has also been demonstrated in sympathetic neurons [25], supporting our observations in SCs in the sciatic nerve.

Taken together, we interpret these results that sciatic nerve SCs respond to nerve transection by activation of c-Jun followed by transcription of the* c-Jun* and* ATF-3* genes. Activation of both MAPK and SAPK pathways in response to nerve injury could therefore be linked to Schwann cell proliferation. In addition, this would also support the theory that the SAPK pathway is linked to Schwann cell survival, as without survival, there would not be any proliferation and thus a possibility of an impaired axonal outgrowth.

## 5. Clinical Implications and Conclusions

The complex signal transduction pathways initiated in cells after a peripheral nerve injury and the interplay between neurons and SCs following such an injury pose a fundamental challenge for research on peripheral nerve injury and repair. One of these challenges is the significant aspect on timing of the nerve repair, which is illustrated by the present and other similar studies showing a dynamic activation in the SCs and neurons. Although mechanisms of the inherent regenerative properties of DRG sensory neurons are pivotal, it is imperative to also study other aspects of injury-induced mechanisms to gain further understanding of other cell types and signalling mechanisms that are involved in peripheral nerve regeneration. The major and novel finding of the present study is the differential regulation of c-Jun activation in SCs of the rat sciatic nerve compared to what has been previously described for DRG sensory neurons. It has been previously discussed that the SAPK signalling pathway could be a potential therapeutic target in peripheral nerve injury and repair due to its response to a wide range of cellular stresses as well as in response to inflammatory mediators, which are also involved in a nerve injury. Furthermore, the latter statement may be relevant in conditions where the nervous system is affected by a disease, such as diabetes, where also an additional nerve injury may be present. This is of crucial importance in view of the expected global increase in diabetes. Complications in the peripheral nervous system are common in diabetes, that is, diabetic neuropathy. An aberrant activation in the SAPK signaling pathway may have implication in normal aging and particularly for the development of Alzheimer's disease [26]. In conclusion, a detailed description and understanding of the delicate mechanisms, such as alterations in the signal transduction pathways after nerve injury, behind survival and proliferation in SCs, and the switch into a regenerative state in neurons is an essential part in our efforts to improve the outcome of nerve repair and reconstruction after a nerve injury. The present data may be another small piece of knowledge in the complicated puzzle that we have to create to solve the clinical problem with an insufficient outcome after nerve injury and repair and reconstruction; thus, a true translational research.

## Figures and Tables

**Table 1:** Primary and secondary antibodies.

Primary antibodies	Manufacturer	Dilution
Rabbit-anti p-ERK #9101	Cell Signalling Technology, USA	1 : 500
Rabbit-anti-c-Jun #9165	Cell Signalling Technology, USA	1 : 200
Rabbit-anti-pc-Jun #9261	Cell Signalling Technology, USA	1 : 200
Rabbit-anti-ATF-3 sc-188	Santa Cruz Biotechnology, USA	1 : 200
Mouse-anti-BrdU M0744	DAKO, Denmark	1 : 50

Secondary antibodies	Manufacturer	Dilution

Goat-anti-rabbit AlexaFluor 488 A11034	Molecular Probes, USA	1 : 500
Goat-anti-mouse AlexaFluor 594 A11032	Molecular Probes, USA	1 : 320
